# Reduced basal ganglia μ-opioid receptor availability in trigeminal neuropathic pain: A pilot study

**DOI:** 10.1186/1744-8069-8-74

**Published:** 2012-09-24

**Authors:** Marcos Fabio DosSantos, Ilkka Kristian Martikainen, Thiago Dias Nascimento, Tiffany M Love, Misty Dawn Deboer, Eric C Maslowski, André Antonio Monteiro, Maurice Borges Vincent, Jon-Kar Zubieta, Alexandre F DaSilva

**Affiliations:** 1 Headache & Orofacial Pain Effort (H.O.P.E.), Department of Biologic and Materials Sciences and MCOHR, School of Dentistry, University of Michigan, Michigan, Ann Arbor, MI 48109-5720, USA; 2 Translational Neuroimaging Laboratory, Molecular and Behavioral Neuroscience Institute (MBNI), University of Michigan, Michigan, Ann Arbor, MI 48109-5720, USA; 3 Faculdade de Medicina, Universidade Federal do Rio de Janeiro, Federal do Rio de Janeiro, Brazil; 4 Faculdade de Odontologia, Universidade Federal do Rio de Janeiro, Federal do Rio de Janeiro, Brazil; 5 UM3D Lab, Digital Media Commons/MLibrary, University of Michigan, Michigan, USA

**Keywords:** Trigeminal Neuropathic Pain, Opioid system, Neuroplasticity, Chronic pain, Positron emission tomography

## Abstract

**Background:**

Although neuroimaging techniques have provided insights into the function of brain regions involved in Trigeminal Neuropathic Pain (TNP) in humans, there is little understanding of the molecular mechanisms affected during the course of this disorder. Understanding these processes is crucial to determine the systems involved in the development and persistence of TNP.

**Findings:**

In this study, we examined the regional μ-opioid receptor (μOR) availability *in vivo* (non-displaceable binding potential BP_ND_) of TNP patients with positron emission tomography (PET) using the μOR selective radioligand [^11^C]carfentanil. Four TNP patients and eight gender and age-matched healthy controls were examined with PET. Patients with TNP showed reduced μOR BP_ND_ in the left nucleus accumbens (NAc), an area known to be involved in pain modulation and reward/aversive behaviors. In addition, the μOR BP_ND_ in the NAc was negatively correlated with the McGill sensory and total pain ratings in the TNP patients.

**Conclusions:**

Our findings give preliminary evidence that the clinical pain in TNP patients can be related to alterations in the endogenous μ-opioid system, rather than only to the peripheral pathology. The decreased availability of μORs found in TNP patients, and its inverse relationship to clinical pain levels, provide insights into the central mechanisms related to this condition. The results also expand our understanding about the impact of chronic pain on the limbic system.

## Introduction

Trigeminal neuropathic pain (TNP) disorders, such as classical, atypical and postherpetic neuralgias, are persistent pain conditions that can be either spontaneous, or elicited by light touch to the face [1]. The fact that the current therapeutic modalities that focus only on peripheral mechanisms (e.g. microvascular decompression and percutaneous stereotactic rhizotomy) do not provide long lasting relief for these frequently treatment-refractory patients raises the possibility that the causes for the chronicity of those debilitating disorders may also be related to central nervous system alterations. In fact, cortical thickness changes were found in TNP patients, which co-localized with functional (de)activation following allodynic stimulation (brush induced pain) [2]. Furthermore, those neuroplastic changes in the TNP patients were confined to cortical systems associated with pain experience and modulation, especially associated with the μ-opioidergic system, arguably one of the mechanisms centrally involved in the regulation of multiple aspects of the pain experience [3].

Studies with positron emission tomography (PET) using non-selective (e.g., ^11^C]diprenorphine) radiotracers or ^11^C]carfentanil, a selective μ-opioid receptor (μOR) radiotracer, have shown reduced opioid receptor availability in chronic pain syndromes such as rheumatoid arthritis [4], neuropathic pain [5], fibromyalgia [6] and complex regional pain syndrome [7]. Such findings might represent either greater occupancy of opioid receptors by their endogenous ligands, down-regulation of opioid receptors after persistent activation during pain, or both [6]. At the present time, it is unknown whether the μ-opioid system is involved in TNP, and the clinical consequences of that involvement. A down-regulation of μORs could explain hypersensitivity (e.g., allodynia, comorbidity with other pain disorders) and frequent treatment refractoriness, including that to opiate medications. Hence, in this preliminary study we investigated changes in the baseline μORs BP_ND_ in patients diagnosed with TNP when compared to age-matched pain-free healthy subjects. Based on the existing literature, we hypothesized that patients suffering from TNP would show reduced μOR BP_ND_ in regions related to pain regulation, possibly representing persistent activation of the endogenous opioid system and subsequent dysregulation due to the ongoing pain. To our knowledge, this is the first study investigating changes in the endogenous μ-opioid system of patients with TNP *in vivo*.

## Materials and methods

### Subjects

We recruited four right-handed refractory TNP patients (three males and one female; mean age = 50.5 ± 16.5), and eight gender and age-matched healthy subjects (six males and two females, mean age = 44.1 ± 14.9). The selection of TNP patients met the criteria defined by the International Headache Society (IHS) [8], American Academy of Orofacial Pain (AAOP) [9] and International Association for the Study of Pain (IASP) Terminology [10]. We only included patients with: A) TNP for at least six months not adequately controlled by previous medicine therapies; B) Minimal average pain score of 4 (moderate to severe) in the visual analogue scale (VAS); C) Unilateral pain; D) Orofacial allodynic region to mechanical (light touch) or thermal stimulation (heat or cold) and E) ages from 18 to 65. The exclusion criteria included: A) Evidence of other local pathology (e.g., orofacial lesion); B) Recent unrelated orofacial surgery or trauma (< 6 months); C) History of systemic disorders (e.g., multiple sclerosis) or D) Chronic pain other than TNP (e.g., back pain or migraine); E) Use of narcotic analgesics (< 6 months); F) Major psychiatric illnesses (current schizophrenia, major depression with suicidal ideation, or substance abuse within two years); and G) Contra-indications to PET. All healthy controls were: right handed; with ages between 18 and 65 years old; with no history of chronic medical illnesses. Approximately 43 subjects applied to participate in the TNP group. However, only four were considered eligible and completed the study. The reasons that determined the exclusion of subjects for this group were: age (1), overweight (9), presence of other chronic pain disorders (18), multiple sclerosis (4) and use of opioid medication (7). Patients in opioid therapy were not recruited for this study. However, the use of other types of medications (e.g. analgesics, anticonvulsants and antidepressants) was not part of the exclusion criteria. This research investigation was carried out in accordance with the bioethical rules for studies involving human beings of the WMA (World Medical Association)––Declaration of Helsinki (1990), and all of the procedures applied were approved by the University of Michigan Investigational Review Board for Human Subject Use, and the Radioactive Drug Research Committee of the US Food and Drug Administration. All subjects gave written informed consent prior to the participation in the study.

### Clinical assessment

**Figure 1:**
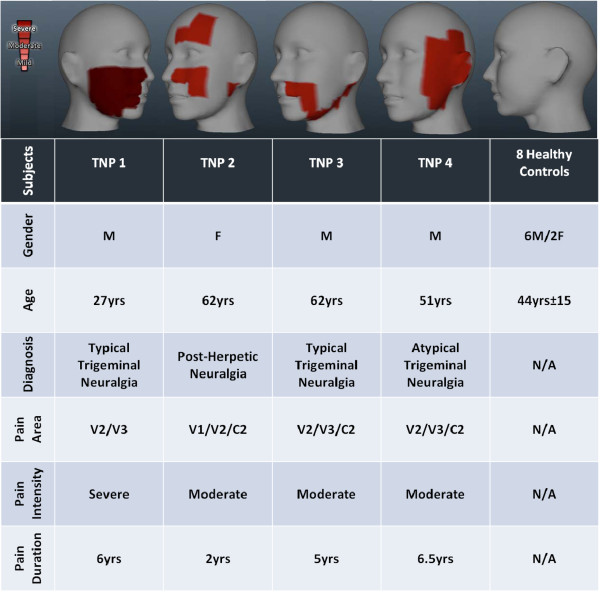
**Clinical profile of Trigeminal Neuropathic Pain patients recruited for this study (for more information, refer to the text).** The pain location was accessed by a craniofacial pain map of the face [12]. This method (PainTrekW, University of Michigan) provides a 3D map of orofacial pain.

All subjects were initially screened by obtaining the medical history, and performing a clinical orofacial pain exam by a pain specialist. During this visit each subject was asked to complete the McGill Pain Questionnaire (MPQ) [11], which provided quantitative measures of clinical pain. This questionnaire has three major classes of pain descriptors (sensory, affective and evaluative), used to measure the subjective pain experience. It also provides the total Pain Rating Index (PRI), based on the rank values of the words selected as descriptors and the Present Pain Intensity (PPI), a 0–5 intensity scale. In addition to the MPQ, patients with TNP were requested to complete an craniofacial pain map [12]. Implemented in an in-house mobile application (*PainTrek*®, University of Michigan), this method provides a 3D head and facial map based on a squared grid system with vertical and horizontal coordinates using anatomical landmarks. Each quadrangle, measuring approximately 1.6 cm × 1.6 cm, frames well-detailed craniofacial and cervical areas and can be filled by the patient to express his/her exact pain location. The method allowed the investigators to precisely localize and measure the total pain area, as well as the dermatomes involved in each TNP patient (Figure 1).

### Neuroimaging

A T1-weighted anatomical MRI scan was acquired on a 3 Tesla scanner (General Electric, Milwaukee, WI). The MRI acquisition utilized the following sequence parameters: axial spoiled-gradient recalled (SPGR) 3D acquisition, 15.63 bandwidth, repetition time [TR] = 9.2 ms, echo time [TE] = 1.9 ms, inversion recovery preparation 500 ms, flip angle = 15°, 25/26 FOV, number of excitations [NEX] = 1, 144 contiguous slices, 1.0 mm slice thickness, 256 x 256 matrix. VBM8 toolbox ( http://dbm.neuro.uni-jena.de/vbm.html) in SPM8 (Wellcome Department of Imaging Neuroscience Group, London, UK) was used for the normalization of the MRI data to MNI (Montreal Neurological Institute) space. PET scans (HR^+^ scanner; Siemens, Knoxville, TN) were acquired in 3D mode (reconstructed full-width/half-maximum resolution 5.5 mm in-plane and 5.0 mm axially), with septa retracted and scatter correction. Tracer quantity ^11^C]carfentanil was administered (10–15 mCi, ≤ 0.03 μg/kg) through an intravenous line (50% in initial bolus and remainder continuously infused to more rapidly achieve constant plasma concentrations). ^11^C]carfentanil was synthesized at high specific activity (> 2000 Ci/mmol) by the reaction of ^11^C]methyliodide and a non-methyl precursor as previously described [13,14]. Images were decay-corrected and reconstructed, and the dynamic frames were coregistered to each other and transformed into tracer transport (K_1_ ratio) and receptor-related (BP_ND_, binding potential) measures. To avoid the need for arterial blood sampling, these measures were calculated using a modified Logan graphical analysis [15], with occipital cortex (a region devoid of μ- opioid receptors) as the reference region. After 5–7 minutes of radiotracer administration, the Logan plot becomes linear with slope = BP_ND_ + 1, which is proportional to μOR concentrations (B_max_)/receptor radiotracer affinity (K_d_) (B_max_/K_d_ ≈ BP_ND_). PET images were co-registered to the individual T1-weighted MRI and then normalized by applying the deformation matrix obtained from the normalization of the T1-weighted MRI data to μOR binding maps.

The control subjects and patients with TNP were compared voxel-by-voxel using unpaired t-test on μOR BP_ND_ data. In view of the small sample size of this pilot study, we set significance at p ≤ 0.001, uncorrected with a priori hypothesis (regions involved in pain modulation). Cluster size is reported for voxels with p < 0.01 in the area of statistical significance.

## Results

No age differences were found between groups (TNP and healthy controls, unpaired t-test, t = 0.676, p = 0.86). TNP patients had average pain duration of 5.4 ± 1.6 years; two patients presented with either spontaneous or evoked pain, while the other two presented both pain modalities. On a scale from 0 to 10 (zero representing no pain and ten representing worst pain possible) the average of spontaneous pain intensity (n = 3) was 5.5 ± 1.3, while the average of evoked pain (n = 3) was 5.4 ± 3.9. All TNP patients had previously tried medications for pain control, including: carbamazepine (subject TNP 3), oxcarbazepine, gabapentin (subjects TNP1 and TNP4), pregabalin (subjects TNP 2 and TNP 4) and amitriptyline (subject TNP 2). Subjects TNP1, TNP2 and TNP3 were under pain control medication during the study. As described before, patients taking opioids were not recruited for this study. The Figure 1 summarizes the main clinical characteristics from each TNP patient included in this study.

**Figure 2:**
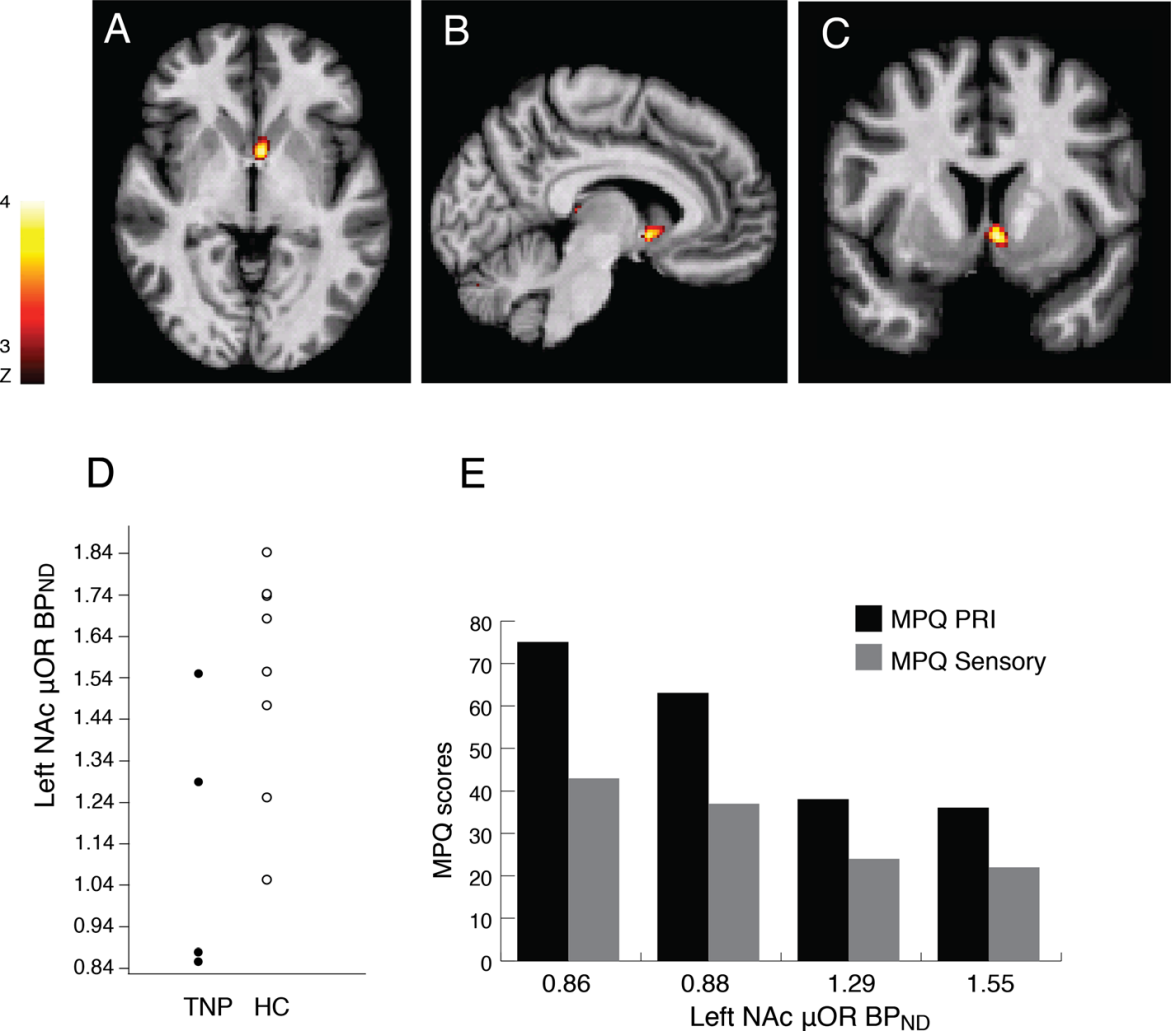
**Changes in the μ-opioid receptor availability in Trigeminal Neuropathic Pain.A**-**C**, Representation of decreased μOR BP_ND_ in the left NAc in axial (**A**), sagittal (**B**) and coronal (**C**) planes (T = 3.2). **D**, Plots of individual μOR BP_ND_ extracted from the left NAc. Each TNP patient is represented in a black circle and each healthy subject in a white circle. E, Correlations between MPQ scores (PRI and Sensory) and μOR BP_ND_ in the left NAc of TNP patients.

TNP patients displayed a significant reduction of the μOR BP_ND_ in the ventral striatal area (z = 2.98, cluster size 989 mm^3^, p = 0.001) of the basal ganglia, with the greatest difference being found in the left nucleus accumbens (NAc) (peak MNI coordinates x/y/z: -4/4/-5). (Figure 2A-D).

The correlation analysis between clinical pain levels and μOR BP_ND_ availability in TNP patients showed that McGill Total PRI scores coincided with reduced μOR in the left NAc. Reduced μOR BP_ND_ in the left NAc was also associated to higher McGill sensory subscale scores (Figure 2E).

## Discussion

The present study compared the μOR BP_ND_ of TNP patients to age-matched healthy controls. We found a significant decrease of μOR BP_ND_ in the basal ganglia, located mainly in the left NAc of patients with TNP, when compared to controls. Other groups have found reductions in opioid receptor BP_ND_ measured with the non-selective radiotracer ^11^C]diprenorphine in other chronic pain disorders, such as rheumatoid arthritis [4], complex regional pain syndrome [7], cluster headache [16] and neuropathic pain [5,17,18]. Those changes involved brain regions known to play important roles in pain processing, such as the insula, anterior cingulate cortex, amygdala, hypothalamus, caudate nucleus and NAc. A significant increase in the volume of distribution of ^11^C]diprenorphine (availability of opioid receptors) was also shown after treatment with radiofrequency thermocoagulation (RFTC) in patients with trigeminal pain [19]. Our findings reinforce the concept that the ongoing pain experience in TNP is linked to the persistent activation of endogenous opioid neurotransmission and the subsequent downregulation of μ-opioid receptors. Considering the limited number of patients recruited for this study, it is possible that increasing the sample size might expand our results to a broader set of brain structures.

In our study, the most significant peak in binding potential reduction of μ-opioid receptors was in the left nucleus accumbens (NAc) of TNP patients when compared to age-matched healthy subjects (Figure 2D). The NAc is located in the ventral striatum, at the interface of sensorimotor and limbic systems, and is part of the circuit involved in the integration of cognitive, affective and motor responses, which is modulated by the endogenous opioid system [20-22]. It receives inputs from limbic areas, such as amygdala and prefrontal cortex and projects to different structures, including brainstem and ventral pallidum [23]. It is largely recognized to be involved in reward and aversive behaviors, and in placebo response [24]. However, there is substantial evidence from both animal [25-27] and clinical studies [3,24,28] that the NAc is involved in pain processing, including TNP [29]. More recently, functional changes in the NAc signal were observed in a model of peripheral nerve injury [27], and a decrease in the NAc gray matter volume was demonstrated in TNP patients [29]. Along similar line, NAc was the only brain region differentiating healthy volunteers and chronic low back pain patients in an fMRI study examining regional brain activations related to acute painful thermal stimulation [30,31]. Regarding its relationship to the endogenous μ-opioid system, the NAc was one of the areas where reductions in μOR BP_ND_ were identified in fibromyalgia patients when compared to healthy controls. Conversely, short- and long-term increases in the μOR BP_ND_ were observed in the same area after traditional chinese acupuncture, which were associated with improvements in clinical pain ratings in fibromyalgia patients [32]. The results of our study support the evidence of the NAc participation in the pain processing, previously proposed in the Motivation-Decision Model of pain****[33,34].

Differences in function of NAc μ-opioid receptors could ultimately contribute to the clinical pain in trigeminal neuropathic pain disorders. A negative relationship was detected between the μOR BP_ND_ in the left NAc and the McGill scores (MPQPRI and MPQ sensory) in sample of four TNP patients (Figure 2E). Subjects with higher McGill scores exhibited lower μOR BP_ND,_ and subjects with lower McGill scores displayed higher μOR BP_ND_. Although the reduced sample size limits the conclusions about this relationship, the results suggest a relationship between the clinical presentation of TNP disorders, and μ-opioid neurotransmission in the NAc, an area related to both pain processing and motivational mechanism. Based on our results, it is possible to hypothesize that the chronicity of trigeminal neuropathic pain is also related to central nervous system molecular neuroplasticity at the level of the μORs in the limbic area of the basal ganglia. This initial proof of concept study supports the initiation of further studies to examine the central molecular mechanisms, such as endogenous opioid neurotransmission, that may influence the clinical course and treatment responses of patients afflicted with persistent TNP.

## Abbreviations

TNP: Trigeminal neuropathic pain; PET: Positron emission tomography; μOR: μ-opioid receptor; μORs BP_ND_: μ-opioid receptor non-displaceable binding potential; NAc: Nucleus accumbens; IHS: International Headache Society; AAOP: American Academy of Orofacial Pain; IASP: International Association for the Study of Pain; VAS: Visual analogue scale; WMA: World Medical Association; MPQ: McGill Pain Questionnaire; PRI: Pain Rating Index; PPI: Present Pain Intensity; MRI: Magnetic resonance imaging; SPGR: Spoiled gradient recalled; TE: Echo time; TR: Repetition time; TI: Inversion time; NEX: Number of excitations; SPM: Statistical Parametric Mapping; MNI: Montreal Neurological Institute.

## Competing interests

The authors declare that they have no competing interests.

## Authors’ contributions

MFD participated in the design of the study, carried out the experiments, analyzed the data, and drafted the manuscript. IKM analyzed the data, participated in the experiments and drafted the manuscript. TDN, MD participated in the experiments. TML participated in the design of the study and data analysis. EM contributed to the methods and illustrations. AAM and MBV participated in the design of the study. JKZ participated in the design of the study and drafted the manuscript. AFD, mentored, conceived, designed, obtained funding, coordinated the study, and participated in the writing of the manuscript. All authors read and approved the final manuscript.
